# The effect of two different GH dosages on final height and bone geometry

**DOI:** 10.1186/s13052-016-0212-4

**Published:** 2016-01-15

**Authors:** Fiorenzo Lupi, Mauro Bozzola, Silvia Longhi, Giovanni Farello, Giorgio Radetti

**Affiliations:** Department of Paediatrics, Regional Hospital, via Lorenz Boehler 5, Bolzano, 39100 Italy; Internal Medicine and Therapeutics Department, Pediatrics and Adolescentology Unit, University of Pavia, Fondazione IRCCS San Matteo, Viale Golgi 19, Pavia, 27100 Italy; Life Health and Environmental Sciences Department, Pediatrics Unit, University of L’Aquila, via Vetoio Coppito, L’Aquila, 67100 Italy; Marienklinik, via Claudia De Medici 2, Bolzano, 39100 Italy

**Keywords:** GH treatment, Bone geometry, Children, Final height, GH dosage

## Abstract

**Background:**

Growth hormone (GH) has a strong positive influence on bone, stimulating both bone elongation and increase in size. The aim of the study was to compare the effect of two different GH dosages on final height and bone geometry in two groups of GH-deficient children.

**Methods:**

We evaluated 121 children (86 m, 35f). Group 1 (77 patients) treated with GH at a mean dose of 0.16 mg/kg/week and group 2 (44 patients) at 0.3 mg/kg/week. Bone geometry was evaluated at final height from a digitalized X-ray of the left hand considering the following parameters: metacarpal index (MI), cross-sectional area (CSA), cortical area (CA) and medullary area (MA).

**Results:**

At baseline, group 2 was shorter than group 1 (−1.54 vs −1.01 SDS; *p* < 0.005), while at final height there was no difference. Height gain was significantly greater in group 2 than in group 1 (1.62 vs 1.13 SDS; *p* < 0.001). Bone geometry: MI was significantly greater in group 2 (0.62 vs 0.55; *p* < 0.001) as well as CA (46.87 vs 42.69 cm^2^; *p* < 0.005), while MA was significantly lower in group 2 (8.48 vs 11.65 cm^2^; *p* < 0.002).

**Conclusion:**

Higher GH doses elicit a significantly greater statural gain and a greater bone cortical area.

## Background

Growth hormone (GH) exerts a strong positive influence on bone by stimulating both bone elongation and increase in size. It enhances the accrual of trabecular [1] and cortical bone [2] up to the attainment of peak bone mass in young adults [3]. GH stimulates longitudinal bone growth and therefore statural growth, acting mainly on the resting zone chondrocytes and inducing local IGF-I production, which stimulates the clonal expansion of proliferating chondrocytes in an autocrine/paracrine manner [4]. In addition, GH increases bone size by IGF-I mediated subperiosteal bone growth [5–9]. We already showed in a previous paper [10] that higher GH doses result in a greater height gain with a trend toward larger bones at final height, leading eventually to improved bone strength. In this study, we wanted to confirm our previous findings by examining a larger group of patients, similarly treated with two different GH dosages.

## Methods

This is a confirmatory study on the effect of two different doses of GH on final height and bone geometry in a large group of GH deficient children. The study was performed in a University clinic and in a Regional Hospital. Final height was measured in both centres by a wall-mounted stadiometer and bone geometry was evaluated on the X-ray obtained for evaluation of bone age. The study protocol followed the ethical principles outlined by the Helsinki Declaration. Adequate information about the protocol supported the decision-making by the parents.

### Subjects

A total of 121 children (86 M and 35 F) affected by isolated idiopathic GH-deficiency (GHD), who reached their final height, were selected for the study. The diagnosis of GHD was based on auxological criteria and a GH peak < 10 μg/l after at least 2 consecutive conventional pharmacological tests. All children had a normal pituitary on MRI, and were not affected by any cardiovascular, respiratory, renal, rheumatic diseases or by any other endocrine disorders. Seventy seven children (57 M, 20 F) age 9.9 ± 3.6 were treated in Pavia with a weekly rGH dosage of 0.16 mg/Kg for a mean period of 6.07 ± 3.2 years (group 1) while forty four children (29 M, 15 F), age 10.3 ± 3.1 were treated in Bolzano with a weekly rGH dosage of 0.3 mg/Kg for a mean period of 5.8 ± 2.7 years (group 2).

The male/female ratio in the two groups was not different (0.66 vs 0.74; NS). They were all regularly followed-up until final height (bone age >15 years in girls and >17 years in boys according to the method of Greulich and Pyle [11]. Height and BMI were converted to standard deviation score (SDS) according to the Italian Standards [12]. However, in order to also take the patient’s genetic potential for growth into account, height was expressed as parentally adjusted height-SDS, i.e. the difference between height-SDS for chronological age and target height–SDS (average height of both parents plus 6.55 cm for boys and minus 6.55 cm for girls). The difference between the parentally adjusted height-SDS on attainment of adult stature and the one before starting treatment was defined as relative height gain. The auxological features of the patients are reported in Table 1 (raw data) and Table 2 (parentally adjusted).

**Table 1:** Clinical data

	Number of subjects	Age at the beginning of treatment (yr)	BMI-SDS at the beginning of treatment	Height SDS at the beginning of treatment	Boys	Girls
Group 1	77	9.89 ± 3.62	−1.5 ± 2.19	−1.76 ± 0.77	57	20
Group 2	44	10.29 ± 3.13	−0.23 ± 1.16	−1.97 ± 0.99	29	15

**Table 2:** Parentally adjusted height SDS and relative height gains

	Height 1 (SDS)	Height 2 (SDS)	GAIN
Group 1	−1.01 ± 0.83^d^	0.11 ± 0.94	1.13 ± 0.70
Group 2	−1.54 ± 1.14^c, a^	0.15 ± 1	1.62 ± 0.69^b^

^a^Group 1 vs Group 2 *p* < 0.005

^b^Group 1 vs Group 2 *p* < 0.001

^c^Height 1 vs Height 2 *p* < 0.001

^d^Height 1 vs Height 2 *p* < 0.01

#### Bone geometry

We used the digitalised X-rays (Dicom files) taken for the assessment of bone age, for the evaluation of bone geometry. The following parameters were evaluated at the level of the 2nd metacarpal bone at its narrowest site, as previously described: outer (D) and inner (d) diameter, metacarpal index (MI = D-d/D: mm), which is a relative measure of the thickness of the 2nd metacarpal cortical bone, total cross sectional area (TCSA: mm^2^), cortical area (CA: mm^2^) and medullary endocortical area (MA: mm^2^). TCSA, CA and MA are not directly measured with this technique, but calculated assuming that the bone is cylindrical. Bone strength was calculated at the metacarpus: it is dependent upon the material property of the metacarpal bones (which cannot be directly measured in vivo) and the cross-sectional moment of inertia which is a function of the fourth power of the cortical ring. By assuming a constant material property, a Bending Breaking Resistance Index (BBRI-1) can be calculated as follows: outer diameter (D) to the fourth power minus inner diameter (d) to the fourth power divided by D [D^4^-d^4^/D]. Furthermore, considering that due to anatomical irregularities, the outer and the inner circumferences are not exactly homogenenous, we tried to overcome this problem by calculating the bone strength using the corresponding areas instead of the diameters (BBRI-2), according to the formula: (CSA^2^-MA^2^)/√CSA).

#### Statistical analysis

The data were normally distributed and are reported as mean ± SD. Student’s paired and unpaired *t*-test were used to verify differences within and between groups, after adjusting for height and sex. Simple correlations were used to investigate the association between the different parameters. A P value of less than 0.05 indicated statistical significance. The SAS Enterprise Guide 4.3statistical software (SAS Institute Inc. Cary, NC, 27513, USA) was used for these analyses.

## Results

*Auxology*: parentally-adjusted height-SDS was significantly higher in group 1 at the beginning of treatment than in group 2 (−1.01 ± 0.83 vs −1.54 ± 1.14; *p* < 0.005), while no difference between the 2 groups (0.11 ± 0.94 vs 0.15 ± 1.0;NS) was observed at the end of treatment. Group 1 (−1.01 ± 0.83 vs 0.11 ± 0.94; *p* < 0.01) and group 2 (−1.54 ± 1.14 vs 0.15 ± 1.0; *p* < 0.001) significantly improved their parentally-adjusted height-SDS, but height gain was significantly higher in group 2 than in group 1 (1.13 ± 0.70 vs 1.62 ± 0.69; *p* < 0.001) (see Table 2).

**Fig. 1:**
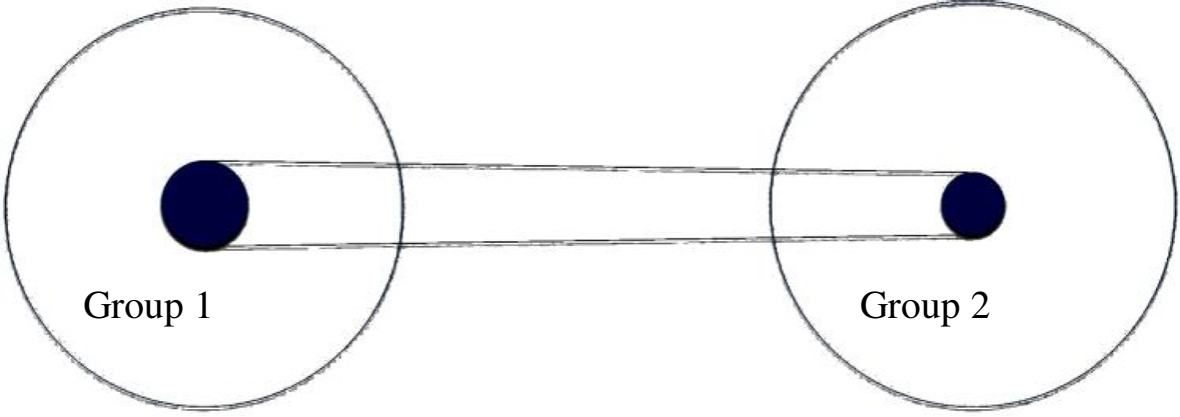
Bone geometry in the two groups

Bone geometry: at final height, group 2 showed a greater MI (0.55 ± 0.07 vs 0.62 ± 0.07; *p* < 0.001), a greater CA (42.69 ± 8.39 vs 46.87 ± 9.39 mm2; *p* < 0.005) but a significantly lower MA (11.65 ± 4.65 vs 8.48 ± 3.87 mm2; *p* < 0.002). There was no difference in total cross sectional area (see Table 3 and Fig. 1). BBRI-1 (556.11 ± 167.69 vs 584.66 ± 177.30 mm^3^; NS), BBRI-2 (389.78 ± 116.57 vs 406.64 ± 123.32 mm^3^; NS).

**Table 3:** Bone geometry and bone strength in the two groups

	MI	CA (mm2)	MA (mm2)	CSA (mm2)	BBRI-1 (mm3)	BBRI-2 (mm3)
Group 1	0.55 ± 0.07	42.69 ± 8.39	11.65 ± 4.65	54.34 ± 11.66	556.11 ± 167.69	389.78 ± 116.57
Group 2	0.62 ± 0.07^a^	46.87 ± 9.39^b^	8.48 ± 3.87^c^	55.35 ± 11.14	584.66 ± 177.30	406.64 ± 123.32

^a^Group 1 vs Group 2 *p* < 0.001

^b^Group 1 vs Group 2 *p* < 0.005

^c^Group 1 vs Group 2 *p* < 0.002

### Correlations

CA was positively correlated with final height (r 0.40, *p* < 0.001), height gain (r 0.22, *p* < 0.05), final BMI, CSA (r 0.92, *p* < 0.0001) and BBRI. MI was positively correlated with BMI at baseline (r 0.38, *p* < 0.0005) and at final height (r 0.39, *p* < 0.0005) and negatively with MA (r −0.82, *p* < 0.001) and CSA (r −0.24, *p* < 0.05). MA was negatively correlated with BMI at baseline (r −0.29, *p* < 0.01) and at final height (r −0.28, *p* < 0.01) and positively with final height (r 0.31, *p* < 0.005) and BBRI (r 0.57, *p* < 0.001).

## Discussion

The major outcome of this study is the confirmation that higher doses of GH elicit a significantly greater statural gain and improvement of the bone geometry. The greater height gain achieved by the children receiving the higher GH dosage is in agreement with previous reports [10, 13–15], emphasizing the advantage of using higher GH doses. Although 0.3 mg/Kg/week is a slightly higher dose than that usually employed in isolated GHD, it is in agreement with the recommendations of the GH Research society [16].

Statural growth is mainly the result of bone elongation under the influence of GH which, moreover, also stimulates the width growth of the bones leading to an increased size. In this study we were able to confirm the positive influence of GH on bone, since the group of children treated more intensively accrued more cortical bone compared to the other group. This finding is also supported by the positive correlations found between cortical area and final height, height gain, cross sectional area and BMI, which are all GH-dependent parameters.

In agreement with our findings, a previous study on adults with growth hormone (GH) deficiency, also showed the strong positive effect of GH treatment on cortical bone, which was exerted by stimulating both periosteal and endosteal bone apposition.

Furthermore, we found a smaller medullary area in group 2 at final height (*p* < 0.002), suggesting that higher dose of GH could induce a clearer effect on the endosteal side (the smaller the area, the clearer the effect) than on the periosteal side of the bone.

MI and CA were therefore significantly higher in group 2 (higher dose GH), strengthening the advantage of using the higher GH dose. However, these geometrical advantages, possibly due to the low number of subjects, were not sufficient to produce a significant improvement in BBRI, although a trend was observed.

One cannot exclude the possibility that another possible explanation for all these geometrical remarks might be that the children in group 2 were significantly shorter at baseline and so, presumably, they also had smaller bones. In this case, the CSA might prove to be increased more efficiently under higher GH dose. Unfortunately, we were not able to calculate the relative gain in the different parameters of bone geometry in the two groups since in many patients the X-rays taken at the beginning of the treatment with GH were not available. Another possible explanation might have been an overrepresentation of females in group 2, since females are known to have a greater endocortical acquisition during puberty. The male/female ratio in the two groups was however not different and differences in sex and height have been taken into account in statistical analysis.

In our study we did not measure for technical reasons bone mineral density at the lumbar spine, which is an important marker of peak bone mass, however, bone geometry measured at metacarpal level is strongly related with bone status in other sites [17–19].

One limit of this paper is the fact that the diagnosis of GHD was based on a level of GH after pharmacological stimulation of less than 10 μg/l while now, in Italy, a value of 8 μg/l is considered as more appropriate. This might have allowed the inclusion of some short normal children, according to the new criteria.

## Conclusion

We confirm the beneficial effects of larger doses of growth hormone on both height gain and bone accretion.

The study protocol followed the ethical principles outlined by the Helsinki Declaration. Adequate information about the protocol supported the decision-making by the parents.
